# Synthesis of Porous and Mechanically Compliant Carbon Aerogels Using Conductive and Structural Additives

**DOI:** 10.3390/gels2010004

**Published:** 2016-01-15

**Authors:** Carlos Macias, Gloria Rasines, Tomas E. García, María C. Zafra, Pedro Lavela, José L. Tirado, Conchi O. Ania

**Affiliations:** 1 I + D Department, Nanoquimia S.L., 14014 Córdoba, Spain; laboratorio@nanoquimia.com; 2 Campus Universitario, IUTA, Universidad de Oviedo, 33203 Gijón, Spain; garciatomas@uniovi.es; 3 Lab Química Inorgánica, University Cordoba, Campus de Rabanales, 14071 Córdoba, Spain; maykazafra@gmail.com (M.C.Z.); iq1lacap@uco.es (P.L.); iq1ticoj@uco.es (J.L.T.); 4 ADPOR Group, Instituto Nacional del Carbón (INCAR, CSIC), Oviedo 33001, Spain

**Keywords:** carbon aerogels, nanoporous, electrically conductive, mechanical properties

## Abstract

We report the synthesis of conductive and mechanically compliant monolithic carbon aerogels prepared by sol-gel polycondensation of melamine-resorcinol-formaldehyde (MRF) mixtures by incorporating diatomite and carbon black additives. The resulting aerogels composites displayed a well-developed porous structure, confirming that the polymerization of the precursors is not impeded in the presence of either additive. The aerogels retained the porous structure after etching off the siliceous additive, indicating adequate cross-linking of the MRF reactants. However, the presence of diatomite caused a significant fall in the pore volumes, accompanied by coarsening of the average pore size (predominance of large mesopores and macropores). The diatomite also prevented structural shrinkage and deformation of the as-prepared monoliths upon densification by carbonization, even after removal of the siliceous framework. The rigid pristine aerogels became more flexible upon incorporation of the diatomite, favoring implementation of binderless monolithic aerogel electrodes.

## 1. Introduction

The flexible coordination chemistry of carbon atoms coupled with recent advances in the field of synthetic chemistry have contributed to the development of highly featured carbon materials with unforeseen control of their physicochemical and structural properties (in terms of particle size and shape, uniform porous void, and so forth), that offer unexpected opportunities in many science and engineering fields beyond traditional uses [1,2,3]. Creative approaches have been reported in the literature in recent years for the synthesis of carbon materials which offer specific benefits compared to traditional ones related to lower costs, greater design flexibility, and high performance [1,2]. Among different strategies, the synthesis of carbon aerogels from the sol-gel polycondensation of resorcinol-formaldehyde mixtures (and some other precursors) has caught the attention of numerous research groups, because of the unique combination of physicochemical and structural properties of the resulting carbon materials, such as controllable pore structure, high electrical conductivity, relatively low cost, and high surface area and porosity [4,5,6,7,8]. Indeed, after the early works of Pekala and coworkers [9,10], the interest for these solids has increased and many research efforts have been devoted to analyze the effect of the synthesis conditions on the morphological and porous structure of carbon gels and consequently on their performance [8,11,12,13].

Carbon aerogels seem particularly promising materials for electrochemical applications as they comply with the requirements typically needed—for instance for energy storage in electrochemical devices (*i.e.*, supercapacitors) or the electro-assisted removal of ionic species from solution (*i.e.*, capacitive deionization)—namely high surface area to form the electric double layer (EDL), resiliency to electrochemical oxidation/reduction, stability under different electrolytes, and so forth [14,15,16].

The technological applications in electrochemistry of carbon aerogels have often been limited by their poor mechanical properties and electrical conductivity [17,18,19,20]. Although nanoporous carbon aerogels typically exhibit higher electrical conductivity than other types of aerogels (which are mostly insulators), it is still a drawback in the case of resorcinol-formaldehyde derived carbon aerogels [21,22]. Hence, despite their *a priori* favorable characteristics—in terms of porosity and resiliency to electrochemical modification—carbon aerogels usually present low current and ionic removal efficiencies [15,16,23,24]. On the other hand, it is well known that the electrical properties of carbon materials are directly related to their structure; most carbon precursors are generally good insulators due to the high ratio of sp^3^ bonded carbon atoms [3,25]. The electrical conductivity of carbon materials increases with the density of conjugated sp^2^ carbon atoms (that may be raised upon thermal treatment of the precursors at temperatures above 1000 °C) and the degree of structural order of the separate conjugated units that interconnect forming a conducting network [3,25]. However, combining high porosity and electrical conductivity is still challenging, and the lack of conductivity of nanoporous carbons is usually overcome by using small amounts of carbon black as low cost conductive additive in the manufacture of the electrodes, to lower the resistance thus enhancing the electrodes performance [23]. Also, the mechanical stiffness of monolithic electrodes is the main reason for the prevalence of the use of electrodes in powder form.

Resorcinol-formaldehyde aerogels are usually fragile materials, although the mechanical stiffness is largely related to the cross-linking degree of the precursors and the porosity of the aerogels [9,20,24]. Organic hydrogels are mechanically reinforced upon carbonization at high temperature to render denser carbon gels, that become more rigid (although typical Young modules are still a factor of 100–1000 lower than those of silica glass) but their deformation capacity decreases [9,20,24]. In a previous study we reported the good electrochemical performance of binderless monolithic aerogel electrodes over powdered materials due to the combination of a nanoporous structure interconnected with a macroporous network [26,27,28]; their technological implementation at large scale was, however, limited due to the large shrinkage and deformation of the pieces after the carbonization; the contact between the bent monolithic electrodes and the current collector was compromised, leading to important efficiency losses due to resistance.

Aiming at fabricating large monolithic electrodes with improved mechanical properties without compromising the porosity and conductivity, we herein report the preparation of carbon aerogel monoliths with enhanced mechanical properties by using low cost structure and conductive additives—diatomaceous earth and carbon black. The synthesis route is a simple modification of the conventional one [9], and consists of allowing the sol-gel polymerization of the precursors (melamine-resorcinol-formaldehyde) in the presence of the additives. Data has shown that the obtained aerogel composites displayed a highly porous structure, and improved electrical conductivity and mechanical properties provided by the additives, even after the removal of the sacrificial siliceous framework. The incorporation of the diatomite during the synthesis prevented the deformation of the electrodes upon densification by carbonization; such mechanical compliance may contribute to the implementation of binderless monolithic aerogel electrodes.

## 2. Results and Discussion

### 2.1. Characterization of the Aerogels

In a previous study we reported the two-step synthesis of N-doped carbon aerogels based on the prepolymerization of melamine-resorcinol-formaldehyde (MRF) mixtures [26,27]. Regardless of the solution pH and M/R molar ratio, the hydrogels prepared by the conventional one-step route displayed essentially a microporous character, as opposed to the meso-/macroporous network of those prepared upon the prepolymerization of the precursors. These MRF aerogels presented promising electrochemical features due to a unique pore structure dominated by large pores, and an improved wettability provided by the presence of N-surface groups. The materials still displayed poor electrical conductivity (although superior to that of RF aerogels) and limited mechanical characteristics (large deformation and bending upon carbonization), for which the use of large monolithic electrodes was quite challenging.

We herein report a simple modification of the above-mentioned method for the synthesis of MRF aerogels consisting of carrying out the sol-gel polymerization of the precursors in the presence of two additives: diatomaceous earth as low cost sacrificial structural additive to improve the stiffness of the aerogels, and a carbon black—commonly used in electrochemical applications—to increase the conductivity of the materials. The diatomite (D) used (Nanolit, K6) is a fine-grained siliceous sediment of biogenic origin (skeletal remains of microscopic single-celled diatoms; average particle size 12 μm; chemical composition: 89.2 wt% SiO_2_, 4 wt% Al_2_O_3_, 1.7 wt% Fe_2_O_3_, 0.5 wt% CaO, 0.3 wt% MgO); the carbon black (B) additive (Superior Graphite Co.; average particle size 6 μm; ash content below 0.05 wt%, volatiles below 0.15wt.%) is characterized by a high electrical conductivity (*ca.* 10 mS/cm). The presence of diatomite and carbon black in the aerogels is indicated by either “D” or “B”, respectively. The diatomite was etched off (using HF), unless otherwise stated.

**Figure 1:**
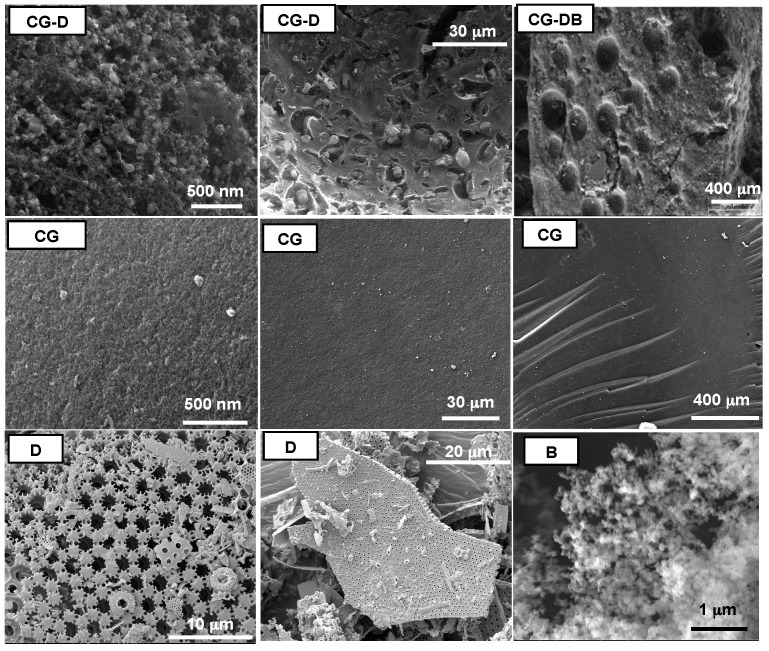
Scanning Electron Microscopy (SEM) images representative of the studied monolithic aerogels prepared in the presence and absence of the additives. Images of the diatomite (sample D) and the carbon black (sample B) are also shown for clarity.

Figure 1 shows SEM images of the carbon aerogels displaying the differences on a macroscopic scale depending on the presence of additives during the synthesis. All the aerogels prepared in the presence of diatomite (samples CG-D and CG-DB) presented similar SEM images, and are characterized by a relatively rough surface with large holes, likely inherited from the siliceous additive.

**Figure 2:**
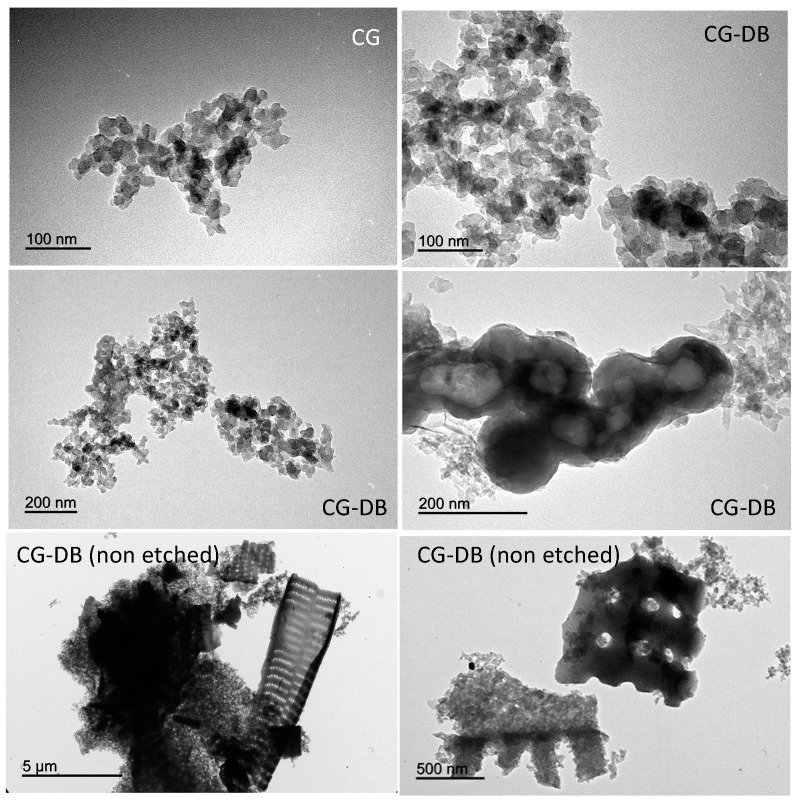
Transmission Electron Microscopy (TEM) images representative of the carbon aerogels showing the presence of the additives.

Conversely, the MRF aerogels displayed a smooth compact surface formed by densely packed spherical particles. This observation is in agreement with the bulk density values of the monoliths compiled in Table 1. While CG and HG materials featured the low density values expected for aerogels, CG-D and CG-DB displayed values close to 0.15 g·cm^−3^, likely due to the large voids created after removal of the diatomite. The presence of the nanometric particles of carbon black can be seen in the TEM images in Figure 2 at different magnifications, where the characteristic spherical aggregates of the carbon black particles (seen also as black spots in the low magnification images) appear distributed within the disordered carbon matrix of the aerogels. The aggregates have varied sizes, and are also seen in the samples before the HF etching treatment.

**Table 1:** Main textural characteristics of the monolithic aerogels obtained from N_2_ adsorption isotherms at −196 °C. Aerogels before (HG) and after (CG) carbonization are compared.

Sample	Bulk Density (g·cm^−3^)	S_BET_^A^ (m^2^·g^−1^)	V_TOTAL_^B^ (cm^3^·g^−1^)	V_MICROPORES_^C^ (cm^3^·g^−1^)	V_MESOPORES_^D^ (cm^3^·g^−1^)
**HG**	0.41	212	1.44	0.07	1.30
**HG-D**	0.64	101	0.58	0.04	0.51
**HG-DB**	0.63	237	0.51	0.05	0.45
**CG**	0.52	522	1.55	0.19	1.30
**CG-D (non etched)**	0.50	230	0.85	0.09	0.74
**CG-D**	0.15	409	1.23	0.16	0.99
**CG-DB (non etched)**	0.53	118	0.41	0.05	0.35
**CG-DB**	0.16	470	0.75	0.17	0.54
**Diatomite**	--	28	0.08	0.01	0.07
**Carbon Black**	--	23	0.17	0.01	0.16

^A^ Apparent surface area evaluated using BET; ^B^ Total Pore Volume measured at p/po ~0.99; ^C^ Micropore volume evaluated using the DR method; ^D^ Mesopore volume evaluated from 2D-NLDFT-HS method.

**Figure 3:**
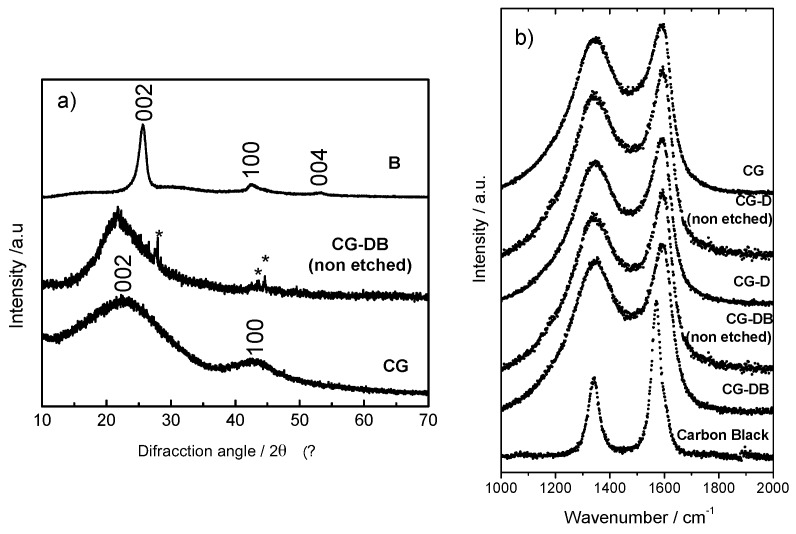
(**a**) X-ray diffraction patterns and (**b**) Raman spectra of the carbon aerogels showing the presence of the additives.

A more detailed analysis of the structure of the carbon aerogels can be inferred from XRD and Raman spectroscopy. The XRD patterns show broad bands at *ca.* 23° and 43° (2θ), characteristic of the (002) and (100) reflections of disordered carbons (Figure 3a) for sample CG. Oppositely, the pattern of the carbon black shows a prompt and narrow peak at *ca.* 25.7° corresponding to the (002) reflection, along with less intense peaks at *ca.* 42.6° and 53.3° evidencing a more ordered structure than the aerogels. Due to the small amount of conductive additive used, peaks due to the presence of the carbon black were not observed in the XRD patterns of sample CG-DB. Similar observations were reported for the series of aerogels loaded with carbon black and subjected to activation under CO_2_ atmosphere to develop the porosity [21,28].

On the other hand, the peaks due to the diatomite (*ca.* 27.7°, 43.4°, and 44.6°) are clearly observed in sample CB-DB non etched (marked with asterisks in Figure 3a). The first-order Raman spectra of the aerogels also showed the typical profiles ascribable to disordered carbons carbonized (Figure 3b). The two broad bands at 1344 cm^−1^ (D band) and 1590 cm^−1^ (G band) are assigned, respectively, to the “disorder-induced” mode upon the lack of long-range translation symmetry and to the “in plane” displacement of carbon atoms in the graphene sheets [29]. The prominent I_D_ band of the carbon aerogels contrasts with the less significant one recorded for the carbon black with a more ordered structure.

The I_D_/I_G_ ratios calculated upon deconvolution of the Raman bands give information about the level of graphitization of the aerogels (Table 2). The samples loaded with carbon black displayed slightly lower I_D_/I_G_ ratio than CG or CG-D (either etched or non-etched), confirming the higher structural order provided by the carbon black. Indeed, the low I_D_/I_G_ ratio obtained for the carbon black corroborated its ordered structure, also in agreement with the XRD patterns.

**Table 2:** Raman parameters (shift and Full Width at Half Maximum, FWHM) for the D and G bands calculated from the deconvoluted spectra.

Sample	I_D_/I_G_	D Shift (cm^−1^)	G Shift (cm^−1^)	D FWHM (cm^−1^)	G FWHM (cm^−1^)
**CG**	2.08	1347.14	1595.57	177.13	72.73
**CG-D (non-etched)**	1.85	1346.80	1597.32	178.01	73.75
**CG-D**	1.95	1346.37	1595.64	179.68	70.96
**CG-DB (non-etched)**	1.76	1346.34	1598.00	181.67	75.06
**CG-DB**	1.82	1347.56	1596.95	169.13	71.37
**B**	0.62	1340.23	1569.63	42.73	35.24

**Figure 4:**
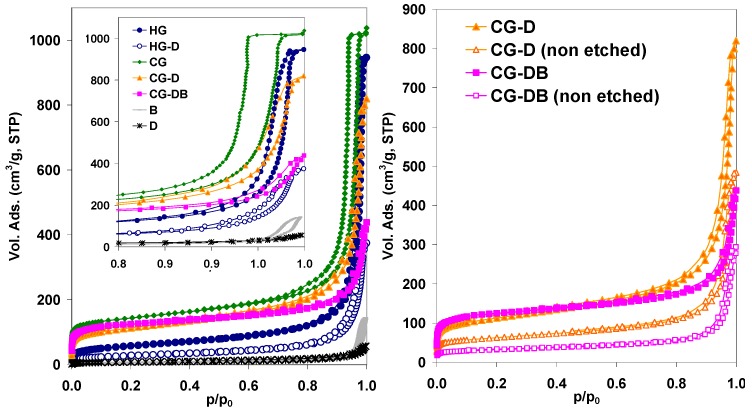
N_2_ adsorption isotherms at −196 °C of the studied aerogels (inset: magnification of the high relative pressure range to show the difference in the hysteresis loops). Data corresponding to the additives (diatomite and carbon black) is also included for clarity.

The effect of the additives in the porosity of the resulting aerogels can be seen in Figure 4 showing the N_2_ adsorption isotherms of the prepared materials; the main textural parameters are also compiled in Table 1. First of all, it is important to highlight that all the materials displayed high porous features, confirming that the incorporation of the additives (either B or D) does not hinder the polycondensation of the reactants [12,18,26,28]. This is outstanding considering the large amount of diatomite used (*ca.* 50 wt%) and the fact that according to the literature, the polycondensation of MRF mixtures is very sensitive to operational parameters such as small pH changes [26]. Comparatively, the amount of carbon black added was smaller (*ca.* 0.9 wt%), and our previous investigations showed that small amounts of this additive do not impede the polymerization of either MRF or RF mixtures [21,26].

Furthermore, all the samples displayed a well-developed porosity with type IVb isotherms according to the new IUPAC classification [30] with prominent hysteresis loops in the desorption branch, characteristic of mesoporous adsorbents. The large adsorbed volume at relative pressures below 0.3 also indicates the presence of a micropore network, characteristic of so-called colloidal aerogels. The presence of D and B during the polymerization of the reactants provoked important changes in the porous features of the resulting aerogels; particularly in the position of the hysteresis loops (hence, mesopore size) and the total pore volumes (Figure 4). It should also be noted that the aerogels retain the porous structure after etching off the siliceous additive (the porous structure does not collapse), confirming that the porosity is due to the adequate cross-linking of the MRF reactants. The large pore volumes and hysteresis loops suggest that the polycondensation of the monomers is slowed down in the presence of the additives (either B or D), as already observed in the case of carbon black alone [21,26,28]. This would lead to a lower degree of cross-linking of the monomers, generating weakly branched clusters that tend to form larger colloidal aggregates in progressively larger pores (pore coarsening) [1,4].

On the other hand, a significant fall in the pore volumes was observed for the samples synthesized in the presence of the diatomite, even after the HF etching (Table 1). This affected the whole range of relative pressures, hence D-containing aerogels displayed lower surface area values than the pristine materials. Otherwise, the addition of carbon black along with the diatomite (sample CG-DB) yielded a decrease in the mesopore volume (compared to CG-D); this contrasts with the trend observed for other composite aerogels prepared when only carbon black was used as additive, which led to an increase in the pore volume in the mesopore range [21,26,28]. When the polycondensation of the reactants is carried out in the presence of both D and B, the formation of large pores is still dominant, which is indicative of the formation of weakly branched clusters during the polymerization reaction; the low pore volumes (compared to D- or B-containing aerogels) suggest that the extent of the assembling of the clusters in large aggregates is partially hindered by the large amount of diatomite.

**Figure 5:**
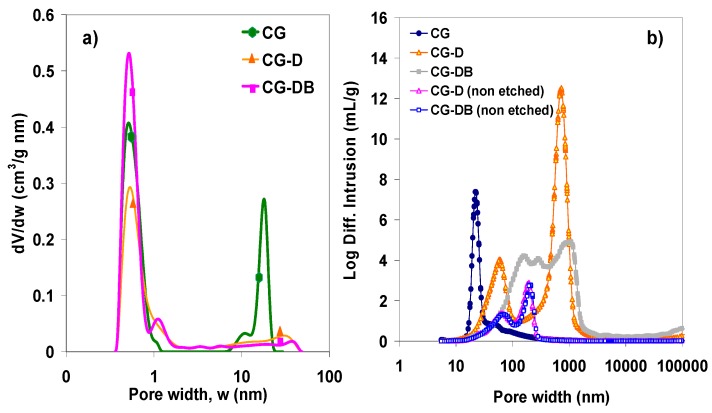
(**a**) Distribution of pore sizes of the carbon aerogels prepared in the presence of diatomite, after removal of the siliceous sacrificial additive obtained by the 2D-NLDFT method applied to the gas adsorption data; (**b**) Distribution of pore sizes in the macropore range obtained from mercury porosimetry.

Further information on the nanotexture of the synthesized aerogels was obtained from the analysis of the pore size distribution. Figure 5a shows the distributions obtained by analysis of the N_2_ adsorption data corresponding to the carbon aerogels after carbonization and removal of the diatomite, as these are the samples of interest for electrochemical applications. In agreement with the shape of the isotherms (*i.e.*, position of the hysteresis loops), the effect of the additives is noticed most remarkably in the distribution of larger pores.

The pristine aerogel (sample CG) displayed the typical monodispersed distribution of pore sizes in the mesopore range, with the average mesopore size around 18 nm [21,22]. In contrast, the composite aerogels treated with diatomite and carbon black displayed a broad distribution in the mesopore range, between 5 nm and 40 nm. Additionally, the incorporation of the diatomite in the synthesis gave rise to the formation of large macropores, as evidenced by mercury porosimetry (Figure 5b), which were not present in the pristine CG carbon aerogel. This clearly shows the capability of the siliceous additive to modify the porous network of the carbon matrix creating large voids in the meso-/macropore range.

### 2.2. Mechanical Properties of the Aerogels

**Figure 6:**
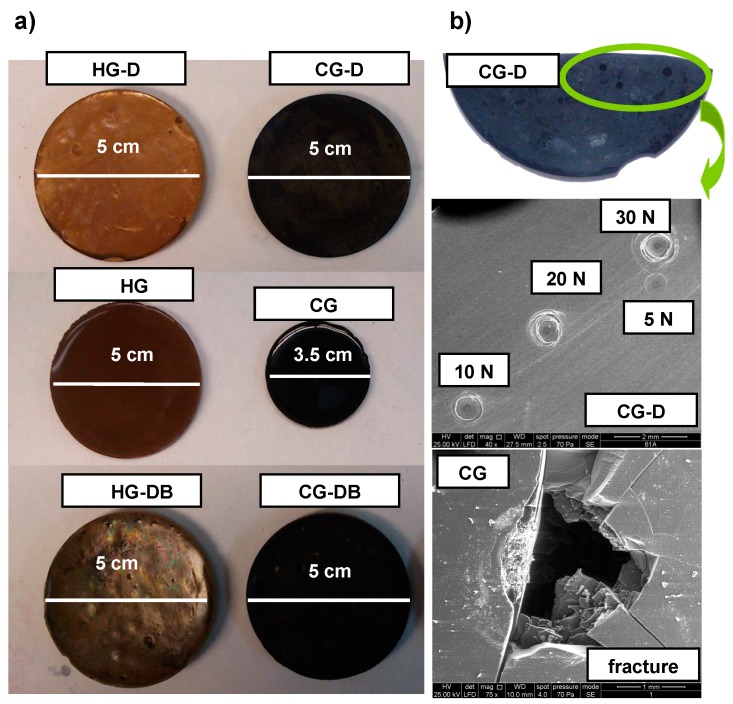
(**a**) Images of the studied monolithic aerogels showing the characteristic shrinkage upon carbonization and (**b**) examples of the characteristic fingerprints of the aerogels after different loadings.

In addition to being highly porous, the aerogels prepared in the presence of the additives were mechanically more compliant. The aerogels ranged from brittle to sponge-like solids when D was incorporated—even after etching off the diatomite—regardless of the presence of the carbon black (Figure 6). The typical shrinkage of the monoliths after carbonization—due to the evolution of volatiles upon densification of the carbon matrix—is shown in Figure 6. Values up to a 30% reduction in volume are typically reached in RF and MRF aerogels (Table 3); this behavior is independent of the composition of the aerogels [27], although it is more pronounced for MRF formulations. For the samples CG-D and CG-DB prepared in the presence of diatomite, the carbonized disks preserved their dimensions, even after the removal of the siliceous skeleton, likely due to the open pore structure of the aerogels provided by the diatomite (Figure 5b), which would facilitate the evolution of the volatiles upon carbonization.

**Table 3:** Shrinkage after carbonization (in C-series) of the aerogels and response to the Crushing Strength Tests. The initial dimensions of all the specimens were *ca.* 5 cm.

Sample	Shrinkage (%)	Rigidity ^§^ (N/mm)	Maximum Load ^§^ (N)	Toughness ^§^ (N·mm)	Behavior on Crushing Strength Tests ^§§^
**HG**	--	324	17	1.2	Brittle, fracture upon load of 21 N
**CG**	30.8	456	28	0.8	Brittle, fracture upon load of 14 N
**CG-D**	5.4	65	23	3.2	Flexible, no fracture after loading at 30 N
**CG-DB**	5.2	27	10	2.9	Flexible, no fracture after loading at 30 N

^§^ calculated from the Load Displacement Curves (see experimental section); ^§§^ evaluated from the Crushing Strength Tests (compression up to 30 N) of the spherical specimens.

These differences in the consistency of the aerogels depending on the use of additives were corroborated by the mechanical tests (performed on the samples of electrochemical interest after etching off the diatomite). First of all we applied a Crushing Strength Test as a screening tool to evaluate the resistance of the materials to fracture upon applying a force up to 30 N [31]. The pristine aerogels (HG and CG) showed the behavior of a rigid material (similar to that of non-porous phenol-formaldehyde resins) with a conchoidal fracture after compression above 30 N (Figure 6, Table 2). Conversely, the aerogels synthesized in the presence of the D followed a different pattern, being gradually deformed with increasing load. The characteristic fingerprint left by the load is shown in the images in Figure 6 for CG-D (a similar behavior was obtained for all the materials prepared in the presence of the D additive); the absence of fracture after 30 N evidences the somewhat flexible character of the aerogels that undergo deformation with the strength without fracture.

**Figure 7:**
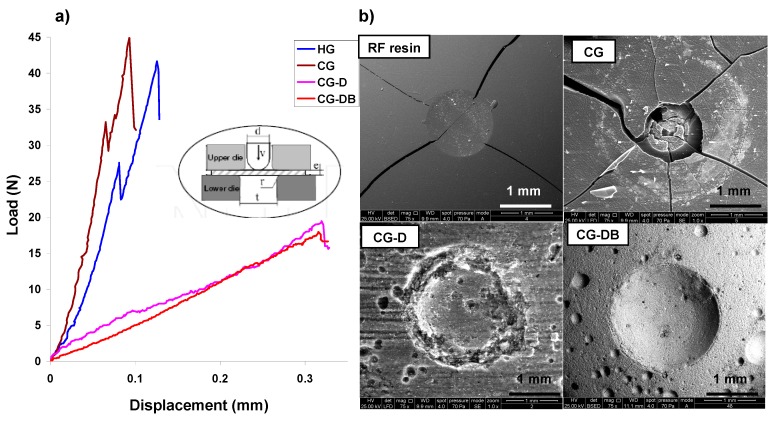
(**a**) Load-displacement curves (LDC) of the aerogels determined by the Small Punch Tests under quasi-statical conditions (inset); (**b**) Characteristic fingerprint of the fracture after the Small Punch Tests on the aerogels (data of a brittle facture on a phenol-formaldehyde resin is also included for comparison purposes).

The mechanical properties were also investigated by means of Small Punch Tests [27,32]. Figure 7 shows the effect of both additives on the load-displacement curves (LDC) from biaxially stretched tests performed on the aerogels. The change from a stiff and brittle behavior of samples HG and CG to a compliant and flexible character of the materials when diatomite was used as additive in the synthesis, is seen in the differences in the shape of the LDC curves. For the aerogels without D, a linear load-displacement behavior with a steep slope is observed, followed by an abrupt drop of the load to zero after reaching the maximum load (associated with pop-in crackings) and leading to a brittle fracture (Figure 7).

When D was used, the LDC profiles exhibit a much more compliant behavior with a smoother slope extending towards higher displacement values before the first crack is recorded, indicating a more flexible behavior than with the corresponding D-free aerogels; this is attributed to the presence of large voids inherited from the siliceous skeleton, as confirmed in the SEM images (Figure 1). The maximum load and rigidity values calculated from the experimental LDC curves at the onset of the first crack and the slope of the LDC curve before the first crack, respectively, are listed in Table 3. The stiffness and deformation capacity of the materials are determined by the maximum load values; as seen, aerogels CG and HG prepared in the absence of diatomite are more rigid (Table 3) than those of CG-D and CG-DB. This is in agreement with the brittle behavior CG and HG observed in the crushing strength tests and the conchoidal fractures upon loading between 14 Nand 21 N (Table 3), as opposed to the compliant character of samples CG-D and CG-DB. The maximum load up to the first pop-in crack is quite similar for all the samples, but CG-D and CG-DB exhibit much higher displacements before the first cracks are observed, indicating their toughness and flexible character.

### 2.3. Electrochemical Characterization of the Aerogels

**Figure 8:**
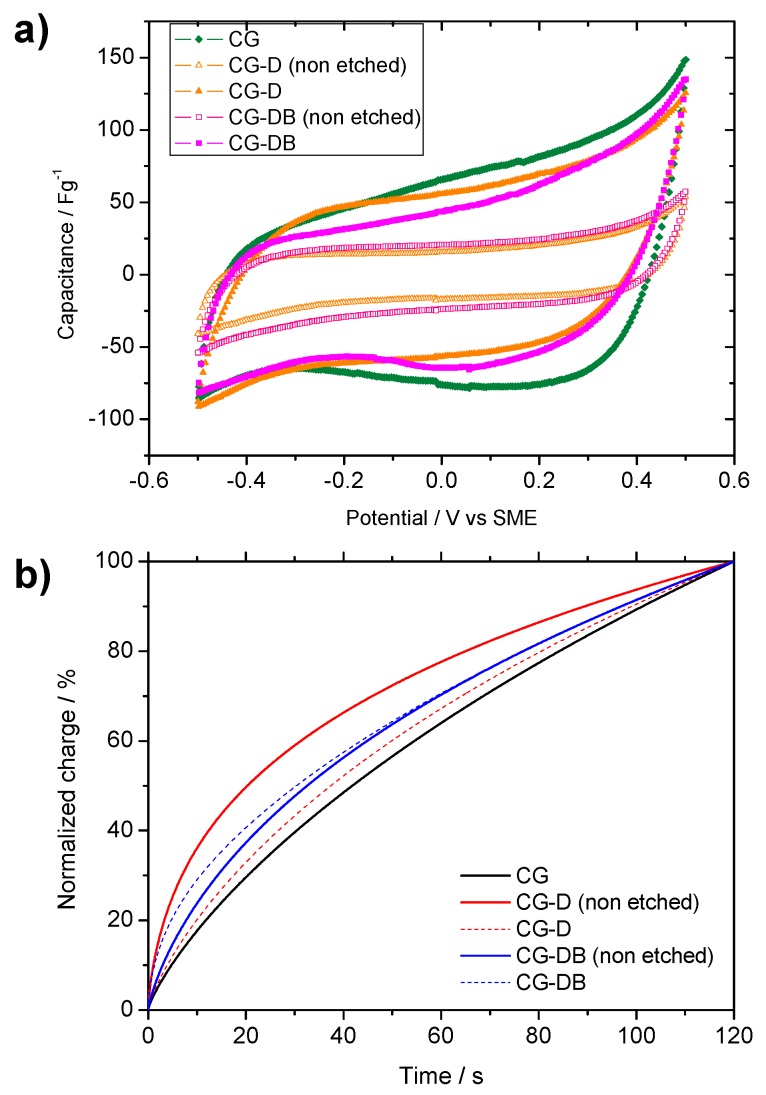
(**a**) Cyclic voltammograms of the studied aerogels recorded at 0.5 mV/s in 0.1 M NaCl; (**b**) Normalized relaxation chronocoulometric curves recorded for the studied samples after a potentiostatic pulse of 300 mV for 120 s.

The electrochemical characterization of the materials was performed by using cyclic voltammetry in 0.1 M NaCl using a 3-electrode cell configuration in a Swagelok-type cell. Figure 8a shows the corresponding voltammograms obtained for the electrodes before and after the removal of the silica additive. As seen, the curves show the box-shape profile characteristic of the capacitive behavior of porous electrodes upon polarization (*cf.* formation of the double layer and adsorption of ions at the electrode surface due to electrostatic interactions). The capacitance value of the aerogel prepared in the absence of additives was 80 ± 3 F·g^−1^; this is the highest value for the studied materials, which is in agreement with the large micropore volume of this sample (Table 1). The effect of the diatomite incorporation in the CG-D sample was to drastically reduce the capacitance value to 20 ± 1 F·g^−1^. This fact agrees well with the textural analysis that revealed a large drop in the specific surface and pore volumes (Table 1). Similarly, a low capacitance was recorded for sample CG-DB (24 ± 1 F·g^−1^) evidencing that diatomite etching is necessary to provide a suitable capacitive response for ion electrosorption. Thus, after etching off the siliceous skeleton (samples CG-D etched and CG-DB etched), the capacitance values increased up to 59 ± 4 F·g^−1^ and 53 ± 3 F·g^−1^. These values are still lower than that recorded for CG aerogel, although correlate well with their differences in porosity. It is evident that the improved macroporosity (at expense of the microporosity) does not lead to an increase of capacitance with respect to the pristine CG (since electrosorption of ions occurs mainly in the micropores). However, it is expected to have a more significant influence on the accessibility of ions from the electrolyte. To unveil this question, the kinetic response of the electrodes was determined by different procedures.

The chronocoulometric relaxation assays after subjecting the electrodes to a potential step of 300 mV *vs.* Hg/Hg_2_SO_4_ for 120 s (Figure 8b) showed fast kinetics in the electrochemical performance (in terms of faster relaxation at short times) for the diatomite-treated samples. This behavior can be explained in terms of the rate of EDL build up and saturation in the samples with different porosity: as seen, samples CG-D and CG-BD non-etched, saturate faster with ions from the electrolyte than the most porous ones.

Impedance spectra allow the determination of the internal resistance of the electrodes to the electroadsorption of ions. By assuming a mixed kinetic and charge transfer control, the kinetic response at the electrode-electrolyte interface can be correlated to both the electrical conductivity and the ion accessibility to inner pores.

**Figure 9:**
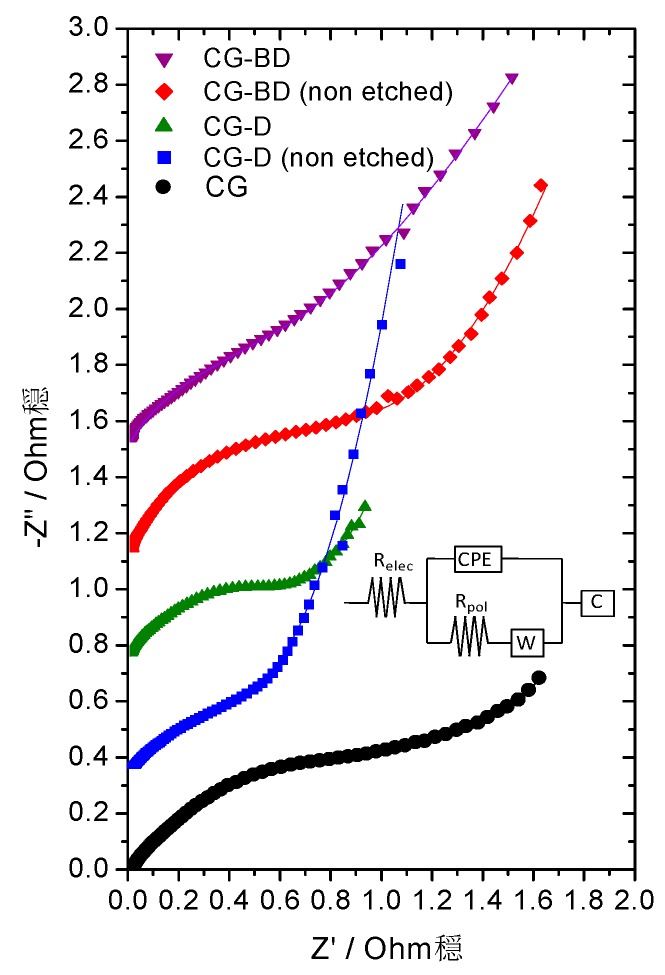
Nyquist plots derived from impedance spectra recorded for the diatomite treated carbon aerogels and carbon aerogel used as a blank sample (for clarity purposes, the curves have been shifted by 0.4 units in the ordinate axis). Inset: Equivalent circuit applied to the fitting of the impedance spectra.

The Nyquist plots recorded for the aerogels are shown in Figure 9; these profiles are largely affected by the polycrystalline and highly porous texture of the electrodes; therefore, numerical fitting to an equivalent circuit is required for quantitative discussion (see inset in Figure 9). The different components of the equivalent circuit used are the electrolyte solution resistance (R_el_), the Warburg impedance (W), a constant phase element (CPE), a capacitor (C), and the polarization resistance (R_pol_). The latter parameter is mainly responsible for the resistance to the ionic migration into the porous structure. The highest R_pol_ value was recorded for the CG sample (1.52 Ohm·g), while an effective decrease in the resistance was observed for the samples still containing diatomite. Thus, R_pol_ values of 0.68 Ohm·g and 1.1 Ohm·g were calculated before etching off the diatomite additive in samples CG-D and CG-DB, respectively. A slight increase in the resistance was observed for sample CG-D (0.74 Ohm·g) after the removal of the additive; we attribute this behavior to an improvement in the accessibility of ions to pores of small sizes (microporosity) that would remain blocked by the additive. For sample CG-DB, the R_pol_ value (0.90 Ohm·g) was slightly better than that of its corresponding non-etched counterpart, pointing out the beneficial effect of carbon black, as a result of enhanced conductivity and the presence of pores of wider sizes (Figure 5).

This was further supported by the electrical conductivity values measured on the monoliths, which are mainly controlled by the presence of carbon black; after etching off the diatomite (insulator), the electrodes presented conductivity values close to those reported for carbon aerogels prepared without siliceous additive, although differences also depend on the formulations (*cf.* N-doped aerogels display higher conductivity than RF ones) [21,26,28]. Values ranged from 2.1 mS/cm for sample CG; 2.2 mS/cm for sample CG-D and 4.3 mS/cm for sample CG-DB.

## 3. Conclusions

We synthesized mechanically compliant monolithic carbon aerogels with improved electrical conductivity by using diatomite and carbon black as structure and conductive additives, respectively. A simple modification of the conventional route for the preparation of melamine-resorcinol-formaldehyde aerogels by sol-gel procedures is proposed based on allowing the polymerization of the reactants in the presence of additives. The resulting aerogel composites displayed a well-developed porous structure, confirming that the polymerization of the precursors is not impeded by the presence of a large amount of additives (*ca.* 50 wt% in the case of diatomite). Furthermore, the aerogels preserved a porous structure after the removal of the siliceous sacrificial additive by HF treatment, confirming that the porosity arises from the cross-linking of the reactants during polycondensation reactions.

The presence of the additives, however, caused a drop in the pore volumes along with the coarsening of the average pore size. Indeed, the aerogels prepared by allowing the polymerization in the presence of the additives are predominantly mesoporous (large mesopores), although microporosity is also developed to some extent (again confirming the adequate polymerization of the MRF monomers in the reactant’s mixture). The effect is more pronounced for the case of D (compared to B), likely due to the large amount used.

Diatomite prevented the structural shrinkage of the as-prepared monoliths upon densification by carbonization, even after etching off the siliceous framework, due to the open pore structure of the additive. The as-prepared monoliths showed the characteristic rigid behavior or MRF aerogels, whereas those obtained in the presence of D displayed a flexible character, with higher maximum loading values and resiliency to fracture upon loads of 30 N. These characteristics evidence that the templating effect of diatomite as anti-shrinkage agent (structural additive), coupled with the presence of low amounts of carbon black as conductive additive, allow the preparation of flexible monolithic carbon aerogel electrodes with a good performance for the electrochemical removal of ions from solution in terms of low polarization resistance and fast electroadsorption. As for the desalting capacity, the development of a large macroporous network of the materials prepared in the presence of the diatomite (at the expense of microporosity) resulted in lower capacitance values. Further work is ongoing aiming at enhancing the capacitance values (via activation).

## 4. Experimental Section

### 4.1. Synthesis of the Aerogels

Hydrogels were synthesized by the polycondensation of Melamine (M), Resorcinol (R) and Formaldehyde (F) using sodium carbonate as catalyst (C) and deionized water (W) as solvent. Carbon black (CB, Superior Graphite Co., Chicago, IL, USA, electrical conductivity *ca.* 10 S/cm) and diatomite (Nanolit K-6, Nanoquimia S.L.) were used as conductive and anti-shrinkage additives, respectively. The molar ratios of the reactants used in the preparation of the gels were (M + R)/C of 135 and (M + R)/W of 0.052. The final pH of the precursors was eventually adjusted to pH 7.4 by adding acetic acid or sodium carbonate, respectively. For the MRF series, the samples were synthesized following a prepolymerization procedure described elsewhere [26]. Briefly, a solution containing R, F, C, and W (solution A) was stirred for 1 h at 40 °C. Separately, a solution containing M, F, W, and C (solution B) was stirred for 30 min at 70 °C. Subsequently, solutions A and B were mixed together and stirred for 20 min at room temperature to further allow the cross-linking of the precursors. Before gelification, diatomite (50% *w*/*v*) and/or carbon black (*ca.* 0.9 wt%) were added and kneaded to ensure a homogeneous dispersion. After the gelation step, a controlled water–acetone exchange was carried out, and the hydrogels were supercritically dried with CO_2_. Finally the aerogels were carbonized under nitrogen atmosphere (*ca.* 800 °C using a heating ramp of 2 °C·min^−1^) to obtain denser carbon aerogels. In the case of the samples incorporating the diatomite, an additional etching off of the siliceous additive was performed using HF, leaving a silica free monolithic carbon aerogel (unless otherwise stated). For the sake of comparison, carbon aerogels prepared by the same synthetic route without the incorporation of the additives were also prepared; as well as samples with the diatomaceous additive before HF etching (labeled as “non-etched”). The nomenclature of the samples is HG for hydrogels before carbonization and CG for the carbonized samples. The presence of Diatomite and Carbon Black in the samples is indicated by adding “D” or “B” to the nomenclature.

### 4.2. Textural and Morphological Characterization

The porosity of the samples was measured by N_2_ adsorption isotherms at −196 °C using a volumetric analyzer (Micromeritics ASAP 2020); the samples were previously outgassed under vacuum (*ca.* 10^−3^ Torr) at 120 °C overnight. For all isotherms, warm and cold free-space correction measurements were performed by using ultrahigh purity He gas (grade 5.0, 99.999% purity).Ultrahigh purity N_2_ (*i.e.*, 99.9992%) was provided by Air Products. The isotherms were used to calculate the specific surface area (S_BET_) and pore volumes (V_total_ and micropore volume, Wo, using the Dubinin–Radushkevich equation). The pore size distributions were calculated by using the new 2D-NLDFT-HS model for carbons with energetically heterogeneous and geometrically corrugated pore walls [33,34] that gave an excellent fit to the experimental data and was free of the common artifacts usually obtained in the PSD analysis when the standard NLDFT model is used. The morphology of the samples was observed by Field Emission Gun Scanning Electron Microscopy (FEG-SEM) with an X-ray Energy-Dispersive System (EDS) in a JEOL JSM-7001F, and in a FE-SEM apparatus (QuantaSEM, FEI), using an accelerating voltage of 25 kV. Transmission electron microscopy (TEM) micrographs were obtained by using a JEOL-JEM2010 instrument. Analyses were performed after the samples were dispersed in acetone. X-ray diffraction (XRD) patterns were recorded on a Siemens D5000 diffractometer equipped with a graphite monochromator and Cu Kα radiation operating at 40 kV and 30 mA. The samples were scanned between 10° and 90° (2θ) at a 0.02°/12 s scan rate. Raman spectra were recorded with a Renishaw Raman instrument (InVia Raman Microscope, UK), equipped with a Leica microscope. The samples were acquired by excitation with a green laser light at 532 nm and the spectra were recorded between 1000 cm^−1^ and 2000 cm^−1^. The spectra were deconvoluted by using Peakfit software package.

### 4.3. Crushing Strength Test

The Crushing Strength Tests were carried out by applying a normal force (up to 30 N) to the as-prepared monoliths. This allows the evaluation of the resistance of the materials to fracture under a compressive strength limit, while measuring the characteristic fingerprint on the material upon deformation and/or fracture [31].

### 4.4. Small Punch Tests

Small Punch Tests were performed under quasi-statical conditions with a low speed tensile test machine on 1 × 1 cm square specimens of 2–3 mm thickness [32]. The specimens were polished to control the thickness and to obtain a flat surface, and framed in a resin framework (1 × 1 mm) surrounding the material while leaving the surface of the material uncovering to allow contact with the punch head. The test consisted of fixing the specimen between two dies (initial load of 2 N), and then deforming the specimen quasi-statically up to failure by means of a small semi-spherical punch with a head of 2 mm of diameter (biaxial expansion). The test is speed controlled with a punching speed of 0.2 mm/min. The displacement of the punch is measured by means of an extensometer, and after correction of the flexibility of the testing device, the displacement of the central point of the specimen is calculated. Thus, the characteristic load displacement curve (LDC) of each material is obtained; this curve represents the force exerted from punch against the specimen (*i.e.*, the load reaction) *vs.* the displacement of the punch. From the profiles, the rigidity (slope of the curve before the first crack), maximum load (onset load of the first crack) and toughness (area under the curves up to maximum load) were evaluated.

### 4.5. Electrochemical Characterization

Cyclic voltammetry measurements were performed in SwagelokTM type cells using a three-electrode configuration. The working electrode was manufactured by spreading on a titanium disk a homogeneous slurry containing the aerogels (70%), carbon black as percolator (20%) and polyvinylidene fluoride binder (10%) in *N*-methyl pyrrolidone. A platinum wire was used as a counter electrode, and Hg/Hg_2_SO_4_ (SME) as reference electrode. Cyclic voltammograms were recorded between −500 mV and +500 mV *vs.* SME at sweep rates ranging from 0.5–10 mV·s^−1^ in a Biologic VMP multichannel potentiostat, in 0.1 M NaCl. Chronocoulometric curves were performed by inducing a potentiostatic pulse of 300 mV *vs.* SME for 120 s and recording the transient current. Impedance spectra (EIS) allowed analyzing the kinetic response of the electrodes to the adsorption reaction; measurements were recorded in an Autolab PGSTAT12 system, using an AC voltage signal of 5 mV *vs.* equilibrium potential over the frequency range of 25 kHz to 10 mHz. The electrical conductivity was measured on previously dried as-prepared monolithic samples; the specimens were packed between two metallic collectors and held under constant pressure and a bias voltage between 0.2 V and 1.2 V was applied (0.2V/s step for 120 s) using an Arbin MSTAT (Arbin Instruments Inc., College Station, TX, USA) potentiostat. The resistance was calculated from the slope of the current-voltage profiles at each voltage.
